# Rheology in Product Development: An Insight into 3D Printing of Hydrogels and Aerogels †

**DOI:** 10.3390/gels9120986

**Published:** 2023-12-17

**Authors:** Raquel V. Barrulas, Marta C. Corvo

**Affiliations:** i3N|Cenimat, Department of Materials Science (DCM), NOVA School of Science and Technology, NOVA University Lisbon, 2829-516 Caparica, Portugal; r.barrulas@campus.fct.unl.pt

**Keywords:** rheology, 3D printing, hydrogels, aerogels

## Abstract

Rheological characterisation plays a crucial role in developing and optimising advanced materials in the form of hydrogels and aerogels, especially if 3D printing technologies are involved. Applications ranging from tissue engineering to environmental remediation require the fine-tuning of such properties. Nonetheless, their complex rheological behaviour presents unique challenges in additive manufacturing. This review outlines the vital rheological parameters that influence the printability of hydrogel and aerogel inks, emphasising the importance of viscosity, yield stress, and viscoelasticity. Furthermore, the article discusses the latest developments in rheological modifiers and printing techniques that enable precise control over material deposition and resolution in 3D printing. By understanding and manipulating the rheological properties of these materials, researchers can explore new possibilities for applications such as biomedicine or nanotechnology. An optimal 3D printing ink requires strong shear-thinning behaviour for smooth extrusion, forming continuous filaments. Favourable thixotropic properties aid viscosity recovery post-printing, and adequate yield stress and *G*′ are crucial for structural integrity, preventing deformation or collapse in printed objects, and ensuring high-fidelity preservation of shapes. This insight into rheology provides tools for the future of material design and manufacturing in the rapidly evolving field of 3D printing of hydrogels and aerogels.

## 1. Introduction

The 3D printing technology is the process of creating 3D structures based on a digital model and has revolutionised several industries, from medicine to construction, with significant implications for product development [1,2,3,4,5]. This article looks at rheology and its central role in producing hydrogels and aerogels using 3D printing technologies. Background information will be provided in the following subsections. Rheology, the science of how materials flow and deform, is crucial in determining these advanced materials’ printability and final properties. By exploring the connection between rheological behaviour and material design, we aim to offer insights that could provide tools for the future of material design and manufacturing.

### 1.1. Overview of Rheological Concepts

Printing is the process of flowing and transferring fluid ink. When the fluid experiences external pressure to induce motion, the cohesive forces between the liquid molecules create an internal frictional force that impedes movement between them. Viscosity can be defined as the quantification of the internal resistance to shear forces within the liquid. The viscosity (*η*, in Pa·s) is commonly described by the ratio of shear stress (Equation (1)), where *F* is the shear force (in N, Newton) and *A* is the shear area (in m^2^). The unit for shear stress is N/m^2^ or Pa (Pascal).

(1) $$\tau =\frac{F}{A}$$

For shear rate (Equation (2)), where *v* is the velocity (in m/s) and *h* is the shear gap (in m), the unit for shear rate is 1/s or s^−1^ [6,7].

(2) $$\dot{\gamma }=\frac{v}{h}$$

**Figure 1:**
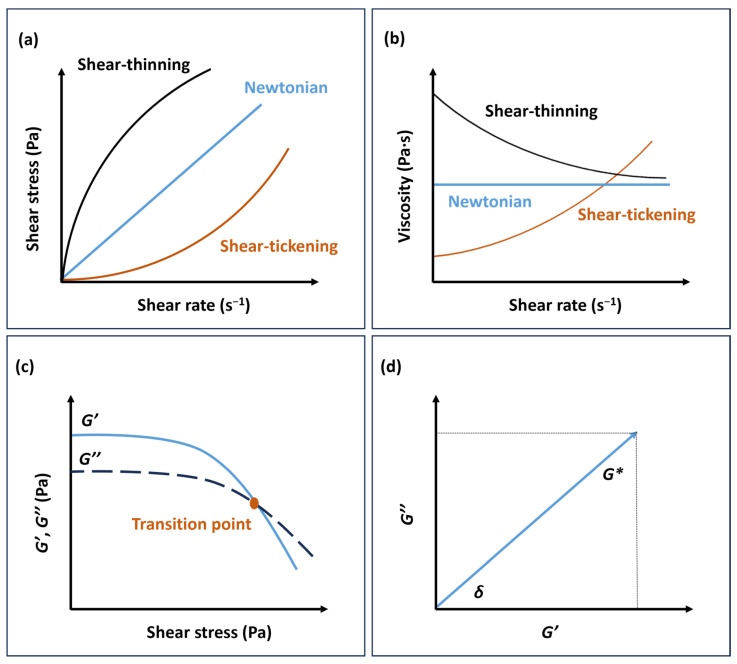
Newtonian and non-Newtonian fluids with (**a**) shear stress versus shear rate and (**b**) viscosity versus shear rate. (**c**) Storage (*G*′) and loss modulus (*G*″) of shear-thinning non-Newtonian fluid versus shear stress. (**d**) Relationship between the complex shear modulus (G*) and *G*′, *G*″.

Viscosity is influenced by factors such as ink concentration, substance type, and temperature, and it can exhibit either a constant value or undergo variations as the shear rate increases. Consequently, fluids can be classified as either Newtonian or non-Newtonian. In the case of Newtonian fluids, the shear stress experienced by the fluid increases proportionally with the shear rate, resulting in a constant viscosity. As a result, Newtonian fluids experience flow under the influence of a minor external force. On the other hand, non-Newtonian fluids exhibit variable viscosity, which can either decrease or increase with an increase in shear rate. Based on this behaviour, they can be categorised as shear-thinning (or pseudoplastic) or shear-thickening (or dilatant) fluids (Figure 1a,b) [7].

The shear-thinning behaviour of fluids is of great significance in the printing process. It is characterised by decreased viscosity as the shear rate increases, ensuring smooth ink flow during printing when subjected to driving forces. Furthermore, these fluids exhibit a certain level of inherent stability, enabling them to preserve the printed shape or pattern even after the external driving force has been eliminated. In contrast, shear-thickening fluids exhibit an increase in viscosity as the shear rate rises. At the same time, they can gradually return to a state where they flow easily when at rest [6,7].

According to the power law, the viscosity of a fluid can be described as a function proportional to the power of the shear rate, represented as Equation (3):

(3) $$\eta =k.{\dot{\gamma }}^{n-1}$$

where *η* is the viscosity, *k* is the consistency factor, *n* is the viscosity exponent, and $\dot{\gamma }$ denotes the shear rate. Newtonian inks exhibit a viscosity exponent value of *n* = 1, whereas inks with shear-thinning behaviour have a value of *n* < 1. Characteristically, the shear-thinning behaviour of ink is often depicted in a plot that illustrates the change in apparent viscosity in relation to the shear rate. A rapid decline in viscosity with increasing shear rate indicates that the ink possesses a stable, solid-like structure at rest, behaving like a fluid as the shear rate rises [8].

Until now, it has been assumed that even for a non-Newtonian fluid, a given shear rate correlates with a given value of shear stress and, hence, viscosity, a value that does not change as long as the value of the shear rate remains constant. However, there are cases where this is different. Thixotropy is the property of certain fluids and gels to become thinner when a continuous force is applied. After reducing the force, the viscosity returns entirely to its initial state within a reasonable time [9]. The higher the applied force, the lower the viscosity becomes. Thixotropy is a time-dependent phenomenon, as the substance’s viscosity must recover after a specific time when the applied force is removed [10]. The opposite behaviour is called non-thixotropy. 

Apart from viscosity, viscoelasticity is a critical factor influencing the printability of inks. To ensure the ink retains its shape after deposition, it must exhibit viscoelastic behaviour and possess an adequate yield point or yield stress. The yield point refers to the minimum force required to surpass the structural integrity of a stationary ink sample, thereby inducing its flow. The complex shear modulus (G*) depends on the shear-stress amplitude and the strain amplitude (Equation (4)) in Pa, where the shear-stress amplitude $\tau_{A}$ is in Pa and the strain amplitude $\gamma_{A}$ is dimensionless or expressed in % and describes the overall viscoelastic behaviour of a sample.

(4) $$G^{*}=\tau_{A}/\gamma_{A}$$

G* can be divided into the storage modulus (*G*′) and the loss modulus (*G*″), which represent the elastic and viscous properties of the ink, respectively (Figure 1c,d) [6,7,8]. 

Before the measurements, the limit of the linea, the linear viscoelastic region (LVER), indicates the range in which the test can be performed without destroying the structure of the sample. For the evaluation, the curve of the *G*′ function is often preferred, as this function has a constant value or plateau in the LVER [6]. 

The elastic modulus, denoted as *G*′, characterises the reversible elastic energy stored during deformation and reflects the material’s elasticity. On the other hand, the viscous modulus, known as *G*″, quantifies the energy dissipated during irreversible viscous deformation. The viscoelastic nature of the material can be inferred by the ratio of the plateau of the storage modulus to the loss modulus (*G*′/*G*″). When the storage modulus significantly surpasses the loss modulus (*G*′ >> *G*″), the material primarily experiences elastic deformation, suggesting a solid-like state. Conversely, when *G*′ << *G*″, a viscous deformation takes place, implying a liquid-like state [7,8]. In the case where *G*′ and *G*″ are comparable, the material exhibits a semi-solid state, typical of gels. The yield stress represents the stress level at which the storage modulus declines with increasing shear stress. It signifies the stress required to induce irreversible plastic deformation and initiate flow in the substance. The point where *G*′ = *G*″ marks the transition from a solid-like state to a liquid-like state. For printing processes involving high viscosity, such as screen printing and extrusion, achieving equilibrium between *G*′ and *G*″ is crucial. The ink’s effective printability in these scenarios relies on maintaining sufficiently high values of *G*′ and *G*″ along with an appropriate yield stress [7,11].

For more details on the basic theory of hydrogel rheology in extrusion 3D printing, consult the review of Herrada-Manchón et al. [12].

### 1.2. Overview of 3D Printing

The 3D printing is an additive manufacturing (AM) process that can be used to produce a variety of structures/geometries using 3D model data, which involves the sequential deposition of layers of materials, one on top of another [13,14]. 

The predominant approach to 3D printing, particularly using polymer filaments, is called fused deposition modelling (FDM) or fused filament fabrication (FFF), which uses a continuous thermoplastic polymer filament to 3D print layers of material. The filament is heated at the nozzle to reach a semi-fluid state and then extruded onto the platform or previously printed layers [11,13,15,16]. Another extrusion-based 3D printing technique is robocasting, direct ink writing (DIW), or simply 3D printing [11,13,16]. 

In addition, the main AM methods are: (a) Selective laser sintering (SLS)—for polymers, metals, and alloy powders; (b) Selective laser melting (SLM)—for metals such as steel and aluminium; (c) Three-dimensional printing (3DP)—method using a liquid binder. The chemical composition and rheological properties of the binder, along with the dimensions and morphology of powder particles, the rate of deposition, the interplay among the powder and binder, and subsequent post-processing techniques, all exert influence in the realm of 3DP; (d) Inkjet printing—ink droplets are ejected in a predefined pattern using thermal or piezoelectric actuators. The applied materials are then polymerised by crosslinking methods such as UV light and chemical and ionic crosslinking [17]; (e) Contour crafting—the primary method of AM for large building structures. This approach can be used to extrude concrete paste or soil employing larger nozzles and high pressure [13,14]; (f) Stereolithography (SLA)—uses UV light (or electron beams) to initiate a chain reaction on a resin layer or a monomer solution. SLA refers specifically to vat photopolymerisation (VP), where photocurable resins are exposed to a UV laser and undergo a chemical reaction to become solid. The chemical reaction involves chemical compounds such as photo-initiators, additives, and reactive monomers/oligomers [13,14,16,18,19]; (g) Direct energy deposition (DED)—an approach that can be used to produce high-performance super-alloys. This uses an energy source (laser or electron beam) focused directly onto a small substrate area to simultaneously melt a starting material (powder or wire). The molten material is then applied to the molten substrate, fused, and solidified after the laser beam has moved; (h) Powder bed fusion—consists of thin layers of fine powders spread on a platform and packed tightly, and a laser beam or a binder fuses the powders in each layer. Successive layers of powder are rolled onto the previous layers and fused until the final 3D object is complete [13,14]; and (i) Laminated object manufacturing (LOM)—the approach relies on the layer-by-layer cutting and laminating of sheets or rolls of materials. Each layer undergoes precise cutting using a mechanical cutter or laser, after which the layers are joined together or, conversely, bonded prior to cutting. Ultrasonic additive manufacturing (UAM) emerges as a recent subset of the LOM method. It integrates ultrasonic metal seam welding with cellulose nanocrystal (CNC) milling throughout the lamination process [13,14,18,19].

Polymers stand as the predominant materials within the 3D printing industry because they are versatile and easily lend themselves to several 3D printing methods. Within AM, polymers manifest in forms such as thermoplastic filaments, reactive monomers, resins, or powders. Bio-fabrication is the process of fabricating tissues and organs through techniques encompassing bioprinting, bio-assembly, and maturation. A fundamental distinction between bio-fabrication and traditional AM lies in the incorporation of cells into the fabricated biomaterials, resulting in the development of bioinks. Bioprinting utilising bioinks integrates methods like laser-induced forward transfer, inkjet printing, and robotic dispensing. Complex structures are designed using Computer-Aided Design (CAD) and transferred to the software that controls the robot. The formulations are then applied layer-by-layer through the nozzle until the desired shape is created [11,13,20]. 

**Figure 2:**
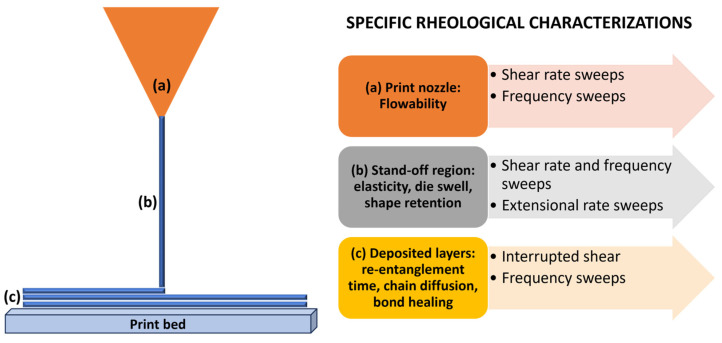
An overview of the relevant rheological characteristics required for depicting the behaviour of a polymer melt. Adapted with permission from Das et al. [21]. Copyright 2021, American Chemical Society.

Let us now take a closer look at the physics of material extrusion. As already mentioned, we must realise that it depends strongly on the melt flow behaviour of the polymers, thereby relying on their rheological properties (Figure 2) [21]. 

In the realm of AM, it is consistently observed that all polymers demonstrate viscoelastic properties, characterised by shear-thinning, shear-thickening, or yield stress fluid behaviour. The successful extrusion of a material, as it pertains to polymer printability and the quality assurance of the printed component, is significantly impacted by the rheological characteristics of the polymeric material [21,22]:

1. Dynamics occurring within the print nozzle. Enhanced shear-thinning characteristics of polymers play a beneficial role in material extrusion-based printing by reducing the requisite extrusion pressure. Maintaining consistent values of *G*′, *G*″, and complex viscosity (η*) in the material extrusion processing range is desirable to ensure a reliable rheological response. This consistency aids in regulating the melt flow behaviour throughout the printing process. When examining fluid flow within the nozzle subjected to heat, one of the foremost parameters is the applied pressure for extruding the molten polymer.

2. Dynamics occurring between the nozzle and print bed (die swell). Shear and compression forces are experienced by the polymer chain network, accumulating residual stress during this process. The viscoelastic nature of the polymer melt gives rise to the generation of normal stresses along both the print direction and perpendicular to it, induced by shear forces [23]. Relaxation of these residual stresses is possible only if the polymer chains are allowed to realign and return to their original state after extrusion through the nozzle. To preserve the shape of the extrudate, the polymer chains must recover from considerable deformation and orientation effects that occur during the extrusion process through the nozzle.

3. Dynamics occurring in the stand-off region between the nozzle and print bed. Low shear rates cause uniform and stable surface characteristics in the extrudates. However, as shear rates increase, sharkskin instability arises, forming extrudates characterised by porous structures and cracks. As shear rates escalate further, stick-slip instability manifests, leading to non-uniform surface features in the extrudate and pressure fluctuations in the nozzle. Lastly, the polymer melt may undergo gross melt fracture instability at extremely high shear rates, distorting the extrudate shape.

4. Rheological measurements pertinent to die swelling (creep recovery and relaxation-stress tests). The extent of stress relaxation can be assessed through the relaxation time, whereby a longer relaxation time corresponds to a slower rate of stress relaxation and a more pronounced elastic response or swelling of the extrudate [24]. Deborah number (*De*), defined as the ratio of the relaxation time scale to the observation time scale during a deformation process, serves as a descriptive parameter aiding in the analysis of die swelling during capillary flow. In the context of the printing process, *De* corresponds to the ratio between the most extended relaxation time scale of the polymer (set on the onset of shear-thinning) and the time required to print a single layer (set on the printing speed). 

5. Post-deposition dynamics on the print bed. Polymer layers experience rapid solidification induced by elevated cooling rates. This phase holds paramount importance as AM parts often exhibit inferior mechanical properties to those manufactured using traditional methods, primarily due to inadequate interlayer adhesion along the perpendicular axis to the print direction. More specifically, weak interlayer bonding can reduce the printed parts’ tensile, compressive, and shear strength perpendicular to the build plane. It makes the parts more susceptible to delamination, especially under mechanical stress. Furthermore, fatigue cracks may initiate and propagate along the interlayer interfaces since this variability in interlayer bonding quality can introduce structural inconsistencies [3,21]. AM has various limitations compared to traditional manufacturing techniques. The speed of AM processes is slower, especially for large-scale production. Although the range of materials available for AM is expanding, it remains limited compared to the materials available in traditional manufacturing. Furthermore, the dimensions of objects that can be produced with AM are often limited by the physical constraints of the 3D printer’s build volume. In addition, AM often incurs higher costs due to post-processing steps and requires skilled labour. Ensuring quality control is a challenge due to the inherent layering effects and material properties [7,21,25]. Therefore, achieving a robust polymer interface necessitates the occurrence of chain rearrangement and surface approach between the polymer chains in adjacent layers, followed by surface wetting, diffusion across the interface, and ultimately equilibration and randomisation. 

6. Post-deposition dynamics on the print bed. Printed parts with enhanced dimensional stability and mechanical strength are achieved when higher values of zero shear viscosity (*η*_0_) are utilised. It is essential for the deposited polymer melt to exhibit elastic behaviour and possess a high yield stress to prevent cracking of the extrudate following extrusion. Furthermore, it is crucial to ensure a rapid transition between the dimensions of *G*′ and *G*″ while under the influence of shear. This guarantees successful extrusion through the nozzle and promotes molecular interdiffusion (*G*″ > *G*′) as well as simultaneously controlling the mechanical robustness and dimensional stability of deposited layers on the print bed (*G*′ > *G*″) [26].

**Figure 3:**
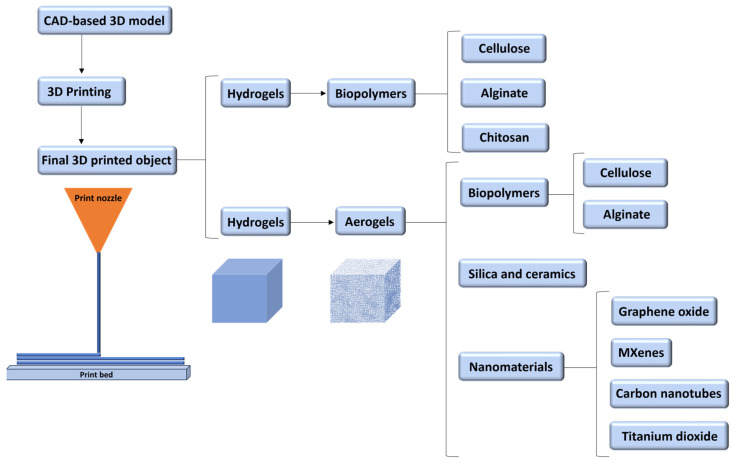
Outline of the materials and products focused on the present work.

Due to the increasing demand for complex and multifunctional products, new materials such as nanomaterials, functional materials, biomaterials, innovative materials, or fast-drying concrete have been explored for 3D printing [18,19]. The present review is summarised in Figure 3, with our primary focus being on the 3D printing of biomaterials.

## 2. 3D Printing of Hydrogels

The use of biopolymers of natural origin as feedstock for 3D printing, such as cellulose, hemicellulose, lignin, starch, alginate, chitosan, and their derivatives, not only meets the requirement for sustainability but also reduces the possibility of negative impacts associated with some synthetic polymers in biomedical applications: degradability, recyclability, harmful degradation products, released additives, and reduced cell attachment. However, they usually need more mechanical strength and durability. Therefore, developing a suitable hydrogel for 3D printing that meets biological requirements and print quality for bio-fabrication is still a significant challenge. The materials should be easily processable and printer-friendly, i.e., have shear-thinning behaviour and high zero shear viscosity, which enable accurate and easy 3D plotting of constructs with fine resolution, high shape fidelity, and structural stability [14,19,22,27,28].

The most commonly used techniques for bioprinting are extrusion-based bioprinting (EBB), material jetting or inkjet bioprinting, and VP. Although both material jetting and VP bioprinting offer higher print resolution than EBB, inkjet bioprinting requires low-viscosity materials (3.00 × 10^−3^–3.00 × 10^−2^ Pa·s), and nozzle clogging is a common problem. In addition, VP bioprinting struggles with the limited choice of photocurable biomaterials and the use of single bio-resin (viscosities of 2.50 × 10^−1^–1.00 × 10^1^ Pa·s). However, EBB is widely used because of its versatility, affordability, scalability, speed, and ease of use. It allows printing a wide range of bioinks with viscosities of 3.00 × 10^−2^–1.07 × 10^−1^ Pa·s and a higher throughput rate than other techniques [29,30,31,32,33,34].

For clarity, the material applied is denoted as biomaterial ink when devoid of living cells within its formulation or as bioink in the case of a formulation containing living cells [35,36].

For EBB, achieving compatibility necessitates that bioinks exhibit adequate rheological properties and employ crosslinking mechanisms conducive to an accurate and precise deposition [37,38,39]. Physically, it is imperative for the material to possess adequate viscosity to be extruded as an independent filament while also possessing ample strength and stiffness to sustain structural integrity post-printing [39,40]. Biocompatibility requires that the materials have suitable swelling properties, are stable for short periods, and are non-cytotoxic to support cell viability [39,41]. Hydrogels typically demonstrate elevated levels of biocompatibility and non-immunogenicity. In these materials, the hydrated network structure fosters an environment favourable to cell encapsulation, rendering them an appealing choice for the formulation of bioinks [39].

The 3D-printed hydrogels mechanical properties are essential to maintaining the structure’s integrity. Increasing the proportion of non-volatile hydrogel components can prevent volume shrinkage and thus preserve the integrity of 3D structures after curing or solidification [42,43]. However, high polymer concentrations within hydrogels may negatively affect cell proliferation, migration, and adhesion [44]. Therefore, regulating hydrogel concentration becomes a crucial element in controlling the effectiveness of 3D-printed items. Paxton et al. presented a straightforward and uncomplicated two-stage screening procedure for developing and formulating bioinks with good printability [45,46].

Ultimately, the optimal parameters for an extrusion-based 3D printer are the conditions under which consistent filaments of the same diameter can be deposited [42,46]. The substrate material employed in the print bed during 3D printing processes is anticipated to influence the quality of the final printed material. The substrate’s properties, including its surface texture and adhesion characteristics related to the hydrophilicity/hydrophobicity of the surface, significantly impact the adhesion of successive printed layers and overall structural integrity [47,48,49,50].

Overall, 3D printing hydrogels enable the production of customisable, complex, and biocompatible structures, mainly advantageous for biomedical and tissue engineering applications. However, there is still limited availability of printable materials for this purpose. Since we are dealing with soft materials, achieving high resolution and printing speed is a challenge that may require additional post-processing steps, such as crosslinking or other treatments. Future developments should focus on improving the mechanical properties, biocompatibility, and printability of hydrogels, as well as understanding long-term in vivo behaviour and the development of efficient post-processing techniques that do not compromise the integrity of printed hydrogels.

Several instances of 3D-printed hydrogels containing biopolymers will be provided to offer additional clarification.

### 2.1. Cellulose

Cellulose stands as the most prevalent natural polymer present on earth. It comprises glucose chains on both the macroscale and the microscale, condensed into fibrils and microfibrils oriented at specific angles to form crystalline and amorphous domains. The most commonly used celluloses are cellulose nanofibrils (CNFs) and cellulose nanocrystals (CNCs) [17,51].

Cellulose and its derivatives, known for their innate mechanical efficiency, biocompatibility, and hygroscopic characteristics, have been acknowledged for their utility in formulating hydrogels and polymer-based composites intended for the production of structural and stimuli-responsive materials through AM processes. The primary advantage of cellulose in 3D printing lies in its elevated aspect ratio, ready availability, processability, sustainability, and suitability for chemical functionalisation. The combination of cellulose with other bio-derived polymers, such as gelatine and alginate, has been extensively explored to formulate hydrogels for 3D printing. Recent advances have also been made in 3D printing cellulose composites with advanced properties using either pure dissolved cellulose or cellulose derivatives such as carboxymethyl cellulose (CMC), methylcellulose (MC), ethyl cellulose (EC), hydroxyethyl cellulose (HEC), hydroxypropyl cellulose (HPC), and hydroxypropyl methylcellulose (HPMC). The broad assortment of cellulose-derived compounds available in various forms, sizes, and characteristics allows for diverse applications of cellulose in 3D printing. These include modifying rheology and acting as a binder, excipient, matrix, or reinforcement [14].

In a study conducted by Heggset et al. [52] in 2019, an examination was undertaken concerning inks designed for 3D printing. These inks were formulated using CNFs individually or in combination with CNCs or alginate derived from various sources. The researchers evaluated the viscoelastic properties of these inks. To enhance stability, alginate-containing biocomposite gels were crosslinked with Ca^2+^. As the solid content of CNF suspensions rose from 3.5 to 4.0 wt.%, the complex viscosities, *G*′ and *G*″, increased as well but reached a plateau with further increases in CNF content. At a concentration of 4.0 wt.%, the complex viscosity at low angular frequency reached approximately 10^4^ Pa·s. This viscosity level was determined to be imperative for maintaining precise shape fidelity in the printed structures of the investigated systems. Substituting a portion of CNFs with CNCs led to a decrease in the complex viscosity, as well as in *G*′ and *G*″. Consequently, this reduction in viscoelastic properties resulted in printed grids with diminished shape fidelity. The alterations observed in the complex viscosity and moduli were contingent upon the proportional variations between CNFs and alginate when the former was replaced by the latter in the formulation. Adding up to 1.5 wt.% alginate as a replacement for CNFs yielded satisfactory shape fidelity. The mechanical characteristics pertaining to the gels’ strength were strongly influenced by the inclusion of alginate and subsequent crosslinking with Ca^2+^. The selection of alginate substantially influenced the mechanical characteristics of the end product but had no impact on the ink’s viscoelastic properties. The results of frequency sweeps conducted at different CNF concentrations were consistent across all the samples examined. They exhibited a higher *G*′ value compared to *G*″, with no crossover point, and a relatively constant to a slight increase in *G*′ and *G*″ with frequency, indicating predominantly elastic behaviour. All the inks based on nanocellulose demonstrated shear-thinning properties. The complex viscosity increased with higher CNF concentrations from 3.5 to 4.0 wt.% and remained relatively constant. The full amount of CNFs and other thickeners remained constant at 4.5 wt.%. Substituting CNFs with increasing concentrations of CNCs led to a decrease in complex viscosity, as CNC particles are substantially shorter and have lower viscosifying properties than CNFs. The resulting properties were quite similar when CNFs were mixed with two different types of alginates. The alterations in complex viscosity resulting from changes in alginate concentration within CNF blends were comparatively restrained, demonstrating smaller deviations compared to those observed in the case of CNCs. However, it appeared that the complex viscosity increased with the addition of a small amount of alginate (0.5 wt.%) but then decreased with higher concentrations. The increase in CNF solid content from 4.0 to 4.5 wt.% did not increase complex viscosity in pure CNF samples. The complex viscosity seems to be primarily influenced by the solid content of CNFs up to 4.0 wt.% and then reaches a plateau. About the strain sweeps, the curves exhibited typical viscoelastic behaviour, with *G*′ higher than *G*″ in the LVER.

In a study conducted by Jiang et al. [53] in 2020, a composite hydrogel of CNC/GEL (cellulose nanocrystals/gelatine) was developed as a biomaterial in 3D printing tissue engineering scaffolds [54,55,56]. The GEL content in the hydrogel samples was consistently 5%, while the CNC content varied. The selected CNC contents (*F_CNC_*) were 0, 5, 10, and 15% (Equation (5)), corresponding to the samples named GEL-5, 5%-CNC/GEL-5, 10%-CNC/GEL-5, and 15%-CNC/GEL-5, respectively.

(5) $$F_{CNC}=\left(\frac{M_{CNC}}{M_{CNC}+M_{GEL}}\right)\times 100\%$$

where *M_CNC_* and *M_Gel_* are the weight content of CNC and GEL in the samples, respectively. All samples exhibited shear-thinning behaviour, with their viscosity decreasing as the shear rate increased. This behaviour was attributed to the disruption of the polymer networks in response to shear stress, resulting in a reduction in viscosity when the disentanglement rate surpassed the network reconstruction rate. The viscosity of the composite hydrogels showed an escalation with increasing CNC content, peaking notably in the case of the 10%-CNC/GEL-5 sample. However, upon surpassing a CNC content of 10%, the viscosity of the composite hydrogel diminished, likely due to CNC agglomeration within the hydrogel system. Furthermore, the viscosity of each sample exhibited a decrease as temperature rose, plausibly due to the heightened kinetic motion of macromolecules at elevated temperatures. This enhanced motion potentially hindered the formation of weak intermolecular bonds among the macromolecules, consequently leading to a reduction in the viscosity of the hydrogel [37]. In terms of viscoelastic properties, all samples exhibit a dependence between both *G*′ and *G*″ and the angular frequency (*ω*). In the composite hydrogels, the presence of CNCs improves the *G*′; however, the *G*′ of the 15%-CNC/GEL-5 sample was lower than that of the 10%-CNC/GEL-5 sample, possibly due to CNC agglomeration. The authors discovered that manipulating the CNC/GEL hydrogel rheological properties through alterations in printing conditions—such as adjusting the nozzle diameter, printing pressure, printing temperature, and nozzle movement speed—had potential to improve the overall quality of the printed filaments. Cell viability and proliferation assays conducted post-crosslinking treatment with genipin, a naturally occurring crosslinker applied at 10 °C for 24 h, proved the biocompatibility of the CNC/GEL scaffolds [28].

More recently, in 2021, several studies were conducted by Wang et al. [57], Tamo et al. [58], and Ma et al. [59]. In summary, Wang et al. [57] reported the development of biomaterial inks using both neutral and negatively surface charged CNFs (mechanically defibrillated CNFs, GrowInk-N, and TEMPO-oxidised CNFs, GrowInk-T) along with photo-crosslinkable biopolymers (gelatine methacryloyl (GelMA) and methacrylated galactoglucomannan (GGMMA)) in Milli-Q water or phosphate buffered saline (PBS) buffer. These inks can crosslink rapidly into a covalent network upon UV irradiation through free-radical chain polymerisation [60]. This crosslinking process can occur immediately after the ink is extruded through the nozzle or while the ink is being dispensed through a particular type of nozzle that allows UV penetration [57]. The formulated inks exhibited shear-thinning behaviour when subjected to shear stress. The viscosity increased for inks with the same composition as the CNF content increased. It was observed that the presence of adsorbed GGMMA prevented CNFs from aggregating into flocs, thereby eliminating hysteresis behaviour and promoting a more uniform microstructure in the GrowInk-N-PBS inks [57]. The rheological properties and printability of the nanocellulose-based inks in light-assisted, hydrogel-extrusion-based 3D printing were primarily determined by the nanoscale dimensions and surface charge of the CNFs when they were used as the main component in combination with a photo-cross-linkable biopolymer. The gel structure of GrowInk-T-based ink formulated with PBS buffer was sensitive to changes in ionic strength, which could complicate its application in cell-laden 3D bioprinting. By adding 2% GGMMA to GrowInk-N-PBS ink, the hysteresis behaviour and uneven microstructure formation of flocs due to variations in ionic strength were significantly reduced, resulting in a decrease in the *G*′ value and flow stress. On the other hand, the addition of 5% GelMA to GrowInk-T increased the flow stress due to electrostatic interactions between GelMA and TEMPO-oxidised CNFs. Inks containing 2% GGMMA exhibited faster crosslinking kinetics and formed stiffer hydrogels than inks with 5% GelMA, owing to the higher degree of methacryloylation and inherent absorption onto nanofibrils. Softer but more elastic hydrogels were obtained with GelMA, which displayed extended strain at break. Adding either GGMMA or GelMA in PBS buffer improved the printability of GrowInk-N-based inks compared to GrowInk-T-based inks when optimising the printing parameters.

Tamo et al. [58] developed 3D-printed hydrogels using chitosan (CHT) and CNFs without chemical modification. The proposed 3D-printed biomaterials, inspired by nature, contained CNF-filled CHT hydrogel. In this biomaterial, CHT acted as a biocompatible framework that facilitated cell growth and had adequate hydrophilic properties, while CNFs reinforced the mechanical properties of the hydrogel made from CHT. The authors utilised EBB to design and develop functional 3D hydrogel scaffolds with low concentrations of CHT (2.0–3.0% *w/v*) and CNFs (0.2–0.4% *w/v*). Flow diagrams were created to illustrate the CNF-filled CHT viscous suspensions and the corresponding CHT solutions. The samples exhibited a steady plateau, indicating Newtonian viscosity. The Newtonian flow region displayed a constant *η*_0_ at low shear rates, suggesting that the shear forces required to untangle the polymer chains were relatively low or comparable to those maintaining their entanglement. However, a shear-thinning behaviour was observed at higher shear rates, with a decrease of *η* as the shear rate increased [61]. Increasing the concentration of CHT led to a higher steady-state shear viscosity of the pure CHT inks. The CHT/CNF viscous inks demonstrated higher Newtonian viscosities within the low shear rate range ($\dot{\gamma }$ < 1 s^−1^). At the same time, shear-thinning occurred in two different regimes, particularly noticeable with higher CNF contents. The possibility of establishing weak electrostatic interactions involving the positively charged CHT and negatively charged CNF surfaces allowed stress transfer from the CHT matrix to the nanofibers. The flow behaviour of the CHT/CNF inks at high shear rates primarily involved disentangling the CHT chains and was minimally affected by the presence of CNFs. This characteristic proved useful for extrusion-based printing, as CNFs could enhance mechanical properties and increase *η*_0_ without significantly affecting the extrudability of CHT-based systems at higher shear rates. By incorporating CNFs, which possessed excellent mechanical properties, into CHT hydrogels, the authors ensured good printability and achieved high-resolution printed constructs without impairing the bioactivity and biocompatibility of CHT. The printable CHT/CNF suspensions (inks) exhibited low viscosities ranging from 100 to 500 Pa·s at a shear rate of 1 s^−1^, enabling the deposition of gel filaments with desirable printing resolution (220–430 µm) and adequate mechanical properties. Ultimately, the utilisation of minimal concentrations of both the biopolymer matrix and nanofiber filler led to the acquisition of durable 3D hydrogel meshes, which facilitated 3D cell colonisation, supported good cell viability, and yielded cellularised bio-constructs.

Ma et al. [59] presented a study on a bio-based ink comprising CNCs and pectin, a biodegradable and cytocompatible polysaccharide known for its high hydration properties [62,63]. The degree of methoxylation (DM) of pectin influences the respective gelation mechanism. High methoxylated pectin (HMP, DM > 50%) forms gels through hydrogen bonding and hydrophobic forces at low pH with high sugar content. On the other hand, with low methoxylated pectin (LMP, DM < 50%), gels are formed by crosslinking the free carboxyl groups with Ca^2+^ ions [64,65]. The study aimed to explore the impact of CNC reinforcement on the 3D printing performance of pectin as a sustainable bio-based material. CNC hydrogels with varying mass fractions (0.5–25 wt.%) were formulated, characterised, and utilised for manufacturing 3D structures. It was found that the viscosity of the hydrogels increased with higher CNC concentrations. At 0.5 wt.% CNC concentration, the hydrogels exhibited shear-thinning behaviour, and at higher concentrations (10–25 wt.% CNC), the hydrogels also showed shear-thinning behaviour because their apparent viscosity decreased significantly as the shear rate increased from 0.01 to 100 s^−1^. This shear-thinning behaviour is advantageous for efficient flow through fine-deposition nozzles. In terms of viscoelastic properties, the results indicated that hydrogels with CNC concentrations above 10 wt.% displayed predominantly elastic behaviour at low shear rates (*G*′ > *G*″) and exhibited well-defined dynamic yield stress (when *G*″ = *G*′). As the shear rates increased beyond the yield stress point, *G*″ became more prominent. Both *G*′ and *G*″ increased with higher CNC concentrations in the matrix. No apparent yield stress was observed for CNC concentrations below 10 wt.%, and the hydrogels showed predominantly viscous behaviour (*G*″ > *G*′), suggesting that these hydrogels were unsuitable for printing. While hydrogels with high CNC loading (10–25 wt.%) were deemed ideal for 3D printing, equipment capable of high printing pressure was required due to their high viscosity and yield stress. The printability results demonstrated that 20 wt.% CNC hydrogels presented optimal print resolution and fidelity, and also that there was CNC alignment along the printing direction. Furthermore, the study revealed that the composite hydrogels produced with both pectins, CNC-HMP and CNC-LMP, displayed favourable rheological properties at the optimal ratio (CNC:HMP = 2:8, CNC:LMP = 10:5), and 3D-printed structures with high fidelity were successfully obtained.

**Figure 4:**
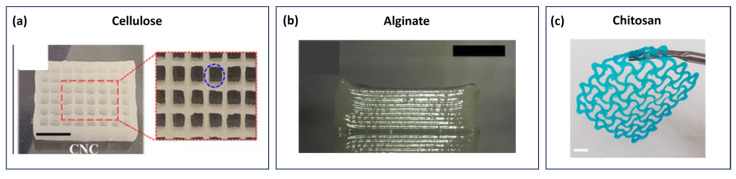
Example of 3D-printed hydrogels from cellulose, alginate, and chitosan, respectively. (**a**) Digital images of the 3D-printed structures with CNC hydrogel plus 50 mM NaHCO_3_. Adapted with permission from Ojagh et al. [66]. Copyright 2023, American Chemical Society. (**b**) The 3D deposition of ALG-Carr-CaSO_4_. Adapted with permission from Kim et al. [77]. Copyright 2019, Elsevier. (**c**) The 3D-printed structure obtained from the TOCNF-chitosan multilayer. Adapted with permission from Ajdary et al. [89]. Copyright 2021, American Chemical Society.

With the same rationale, in 2023, Ojagh et al. [66] detailed the synthesis of electrostatically stabilised nanocrystalline cellulose (ENCC)-based printable hybrid hydrogels. These hybrid hydrogels, formed by combining ENCC with CNC, demonstrated the capability to undergo gelation in the presence of a salt (NaHCO_3_) (Figure 4a). The printability of these hybrid hydrogels was modulated by adjusting the ratio of ENCC to CNC. To render hybrid hydrogels suitable for DIW, they should exhibit shear-thinning behaviour. This property ensures a smooth flow through the narrow printing nozzles. Furthermore, the hybrid hydrogels must recover their gel-like behaviour spontaneously after deposition on the substrate [67]. The attainment of high printing fidelity was accomplished through fine-tuning the rheological properties of the hybrid hydrogels.

In 2023, Gianciosi et al. [68] introduced a novel biomaterial ink tailored for DIW, facilitating the fabrication of intricate 3D multi-graded structures designed for biomedical purposes. This innovative ink formulation integrated multifunctional CNFs, allyl-functionalised gelatin (gelAGE) (0.8–2.0 wt.%), and polyethylene glycol dithiol (PEG(-SH)_2_) (3.0–7.5 wt.%). The CNFs, a rheology modifier, were incorporated at a concentration of 1.8% *w/v* in the inks to optimise printability and retain shape fidelity. Furthermore, the functionalisation of CNFs with azido groups enabled the spatial dispersion of functional components within the 3D structure. Oscillatory tests were conducted to assess the *G*′ of the CNF-based biomaterial ink, accompanied by UV-vis irradiation, which exhibited a gradual increase in *G*′. This increment was corroborated by a reduction in the loss factor (Equation (6)), and the observed buffer time before the elevation of *G*′ suggested a minor interference with the photocrosslinking process attributable to CNFs.

(6) tan⁡δ=G″/G′

In 2023, Tuladhar et al. [69] presented findings on hybrid hydrogels of TEMPO-mediated nano-fibrillated cellulose (TO-NFC), CMC, and alginate (ALG). They prepared compositions, varying TO-NFC concentrations (0.005% to 1.0% *w/v*), 1–4% *w/v* of CMC, and 1–4% *w/v* of ALG, assessing shear-thinning behaviour (described by *n* and *k* values from the power-law model) and its correlation with 3D printability. All compositions exhibited shear-thinning behaviour at 0.005% *w/v* TO-NFC, indicating higher TO-NFC concentrations corresponded to increased viscosity when CMC and ALG concentrations were constant. Changes from 0.005% to 0.01% *w/v* or 0.5% to 1.0% *w/v* TO-NFC had a minor impact on viscosity compared to 0.005% to 0.5% *w/v*. Values of *n* < 1 indicated shear-thinning properties in all compositions. The *k* value, when TO-NFC and ALG concentrations were fixed, highlighted CMC’s role as a viscosity thickener, affecting viscosity significantly. Increasing TO-NFC from 0.01% to 1.0% *w/v* transitioned the physical state from liquid-like to solid-like, where *G*′ became dominant over *G*″, resulting in tan *δ* < 1. Compositions with higher *G*′ values before intersecting with *G*″ exhibited superior mechanical strength due to a more solid-like suspension structure. All compositions were 3D-printable, with most filaments retaining their intended shapes.

### 2.2. Alginate

ALG has demonstrated great potential due to its biocompatibility, biodegradability, and ability to gel with divalent ions [70,71,72]. Its printability can be easily modified by altering the polymer density and crosslinking by adding calcium chloride (CaCl_2_) [39,40,73,74].

However, ALG alone lacks desirable mechanical properties, long-term stability, and functional moieties to enhance cell adhesion and proliferation, which are crucial for bone tissue engineering (BTE). Hence, hydrogel ink formulations of ALG and other materials have been developed to obtain sufficient mechanical properties and biological activity [75]. Therefore, it is necessary to combine ALG with other functional polymers/nanomaterials to get a hydrogel ink whose properties can be tuned on demand by simply adjusting the ratio of different ink components [36]. By adding GEL to ALG, cell adhesion and differentiation can be facilitated, and the viscosity of the hydrogel can be adjusted to meet extrusion and printing requirements [39,40,41].

In 2018, Giuseppe et al. [39] studied a blend of 7% *w/v* ALG and 8% *w/v* GEL to improve printability and compressive modulus. Additionally, the mixture’s viscosity would be low enough to avoid high printing pressures to allow extrusion since high pressures can induce cell death. The increase in compressive modulus with increasing constituent concentrations suggests that the density of ALG and GEL polymers increases hydrogel strength. However, the rapid decrease in modulus over time and the high salt content in the GEL dissolution medium (PBS) made the ALG-GEL blend unsuitable for in vivo applications that experience stresses and strains. The collapse of the network structure due to salt content reduction further contributed to its limitations [76].

In another study in 2019, Kim et al. [77] reported ALG/carrageenan (Carr) composite scaffolds using EBB. A frequency sweep test was carried out with different concentrations of CaSO_4_ in a 2% *w/v* ALG solution to characterise the influence of CaSO_4_ on the mechanical strength of the hydrogel. At the angular frequency of 1 rad/s, *G*′ of the ALG hydrogel increased until 1% *w/v* CaSO_4_ concentration and then decreased. Thus, 1% *w/v* CaSO_4_ was selected for further experiments with 2% *w/v* ALG solution, and by increasing Carr concentration, the viscosity and shear modulus of the ALG-CaSO_4_ hydrogel improved without sacrificing shear-thinning properties. The high thixotropic property of the hydrogel enabled its use in EBB, maintaining the layer-by-layer structure during printing. The ALG-Carr-CaSO_4_ composite (Figure 4b) exhibited excellent structural strength and printability without negatively affecting cell viability. Overall, the combination of ALG with other materials, such as GEL and Carr, has shown promising results in enhancing the mechanical properties and printability of ALG-based bioinks for tissue engineering applications.

In a study conducted by Adhikari et al. [78] in 2021, a biomaterial ink based on ALG/CHT/hydroxyapatite (HA) was synthesised. The polyanionic ALG and polycationic CHT interactions formed a physical gel through ionic interactions. The dispersion of HA, a nanofiller, was achieved using ultrasonication, and its stability in the hydrogel system was ensured through hydrogen bonding with CHT. Post-printing crosslinking was performed using a 10% *w/v* aqueous CaCl_2_ solution. Rheological studies of the printable ink with HA demonstrated shear-thinning properties and an appropriate viscosity, which are essential for EBB. On the other hand, the biomaterial ink comprising only ALG and CHT also exhibited shear-thinning behaviour but had lower working viscosity, resulting in poor printability. The power-law model was used to analyse the shear-thinning behaviour, and it was found that the flow index (*n*) remained below 1 for all the hydrogels, indicating an increase in shear-thinning properties with an increment in ALG concentration. The addition of HA did not disrupt the shear-thinning behaviour of the hydrogel and suggested proper interaction between the components of the hydrogels. Oscillatory strain sweep tests revealed that *G*″ exceeded *G*′ at high strains beyond their LVER, indicating shear yielding and a fluid-like behaviour of the biomaterial ink rather than a solid-like behaviour [79].

This facilitated the extrusion of the hydrogel from the nozzle head in the 3D bioprinter. The inclusion of HA in the hydrogel showed acceptable structural stability after printing; however, concentrations beyond 0.2% by weight of HA resulted in brittleness in the final structure. The ink composition of 5% ALG, 2% CHT, and 0.1% HA (by weight) was identified as a promising combination for tissue engineering applications based on rheological and printability studies.

Recently, in 2022, Sosa et al. [80] conducted a study on optimising ALG–cellulose formulations for use as printable inks. The rapid gelation, adjustable mechanical properties, and non-toxic nature of ALG motivated the researchers’ choice as the main component of the scaffold. Microcrystalline cellulose (MCC) was incorporated to modulate the hydrogel’s mechanical properties for printing purposes. Furthermore, Fmoc-FFY (Fmoc: 9-fluorenylmethoxycarbonyl; F: phenylalanine; Y: tyrosine), a self-assembled peptide known for promoting cell adhesion, was included in the ink without affecting its rheological properties or shear-thinning behaviour. The viscosity of the pre-crosslinked ALG inks was assessed using rheology at different alginate/CaCl_2_ ratios. All ink formulations exhibited shear-thinning behaviour, with viscosity decreasing with the shear rate. By fitting the data to the power-law model, it was determined that inks with a higher amount of pre-crosslinker solution had higher viscosities, as indicated by a more significant value of the parameter *k*. These inks also demonstrated enhanced shear-thinning behaviour, characterised by a lower value of the parameter *n*. Based on these findings, the volumetric ratio of ALG (14% *w/v*) to CaCl_2_ was selected as 1.5:1 for the subsequent 3D printing experiments. After adding MCC, the ink formulation consisting of ALG (8% *w/v*, with PBS addition) and MCC_40_ (40% *w/v*) was chosen for further 3D printing tests due to its improved shape retention capabilities over time. The self-healing properties of the printed scaffolds were evaluated through dynamic step strain amplitude tests, where the strain was varied between 0.1% and 100% over short periods (200 s). At low strains (0.1%), the inks exhibited the characteristic rheological behaviour of a gel (*G*′ > *G*″). However, upon applying a high strain (100%), *G*′ significantly decreased, indicating a transition to a liquid-like state (*G*″ > *G*′). The gel-like behaviour was restored when the samples were subjected to a low strain (0.1%). Frequency sweeps conducted at a strain of 0.1% in the LVER revealed that both ink samples exhibited *G*′ higher than *G*″, confirming their gel-like rheological behaviour. The 3D scaffolds printed using the optimised ink formulation exhibited high shape fidelity. Furthermore, cytotoxicity assessments demonstrated the excellent biocompatibility of the printed scaffolds, highlighting their potential for BTE applications.

In 2022, Nagahara et al. [81] focused on developing a hydrogel-based formulation using ALG and xanthan gum (X). The goal was to create a cost-effective, easily printable bioink with improved mechanical properties. The combination of ALG and X provided several advantages for the formulation. Xanthan gum exhibited shear-thinning behaviour, which facilitated the printing process. Additionally, xanthan gum forms viscoelastic solutions even at low concentrations, making it suitable for bioink applications [82]. The crosslinking of ALG was achieved using strontium ions (Sr^2+^) instead of calcium ions (Ca^2+^). This choice was based on the higher affinity of ALG′s G residues for strontium ions. This led to a more stable and mechanically robust gel than ALG gels crosslinked with calcium ions [83]. Among the various formulations tested, the ALG1.0:X2.5 formulation, consisting of 1% (*w/v*) ALG and 2.5% (*w/v*) X, showed the most promising results. It exhibited shear-thinning solid behaviour, higher yield stress, adequate elastic behaviour, and satisfactory viscosity recovery after exposure to high shear rates. This formulation was considered suitable for 3D printing. Furthermore, the sterile formulation (ALG1.0:X2.5-S) remained suitable for printing even after steam heat sterilisation. This sterilisation method ensured the bioink’s safety while preserving its printability. The sterilised bioink was then used to print a 3D structure resembling the extracellular matrix (ECM), demonstrating its potential for tissue engineering applications.

### 2.3. Chitosan

CHT is soft and cannot support its structure, leading to collapse or deformation under its weight. CHT can be combined with other polymers to address this limitation and enable physical and/or chemical crosslinking [19,84,85,86,87,88,89].

Heidenreich et al. [90] (2020) conducted a study on the rheology of hydrogel precursors containing CHT and collagen (COL) (0.50–1.50% *w/v* CHT: 0.18–0.54% *w/v* COL). The blends of COL and CHT exhibited shear-thinning behaviour, with viscosity values ranging from 0.35 to 2.80 Pa·s at low shear rates. Considering the strain rate associated with 3D bioprinting, the viscosity of the precursors during extrusion ranged from 0.5 to 0.8 Pa·s. Based on suitable viscosity, printability, and polymer ratio, a COL:CHI blend at a ratio of 0.36:1.00% *w/v* was selected to print mono-layered scaffolds for tissue engineering. The hydrogels were formed through nebulisation with NaHCO_3_ and subsequent incubation at 37 °C. NHS/EDC (N-hydroxysuccinimide/1-ethyl-3-(3-dimethylaminopropyl) carbodiimide) was added to the scaffolds to improve their mechanical properties. Both COL and CHT displayed pseudo-thinning behaviour in diluted solutions [91]. The viscosity range of 0.30–30.00 Pa·s was considered suitable for extrusion, as higher values would lead to unstable extrusion due to excessive pressure [92]. Rheological evaluation of the hydrogel precursors demonstrated shear-thinning behaviour and low apparent viscosities (0.35–2.80 Pa·s). The COL:CHT blend at a ratio of 0.36:1.00 was chosen as a biomaterial for 3D constructs in tissue engineering. Adding NHS/EDC improved the mechanical properties of the final constructs. Despite the solubility concerns of the precursors at acidic pH, the researchers achieved a final construct with a neutral pH through nebulisation, resulting in mono-layered scaffolds suitable for cell seeding [28].

Zarandona et al. [88] (2021) investigated CHT hydrogels reinforced with pectin (PEC) and their rheological properties. The binary systems containing both biopolymers exhibited a weak gel-like behaviour, with *G*′ higher than *G*″, indicating proper stability for maintaining dimensional firmness during deposition [93,94]. Stress sweep tests revealed that the hydrogel network with CHT and PEC had lower deformation capacity than single-component systems, suggesting interactions between CHT and PEC [95]. The CHT/PEC systems exhibited shear-thinning flow properties, were favourable for 3D printing, and maintained geometry and mechanical integrity after printing at room or physiological temperatures. The CHT2PEC2 hydrogel was identified as the optimal system for 3D printing due to physical interactions between CHT and PEC, resulting in high cohesiveness and shape retention. The scaffold derived from this system showed potential for biomedical applications such as wound dressing, with a high swelling capacity for wound exudate absorption and good mechanical strength to maintain shape and size after compression sweeps.

In a separate study conducted in 2022 by Ajdary et al. [89], a 3D printed structure composed of nanocellulose (2 wt.%) and CHT (2 wt.%) (Figure 4c) was developed as a substitute for synthetic meshes used in hernia and gynaecological pelvic disorders. The structure relied on electrostatic interactions achieved through layer-by-layer assembly of anionic 2,2,6,6-tetramethylpiperidin-1-yl)oxidanyl (TEMPO)-oxidised cellulose nanofibrils (T-CNF) and positively charged CHT. The biomaterial exhibited shear-thinning rheological behaviour, high printing fidelity, and good biocompatibility. Post-treatment involving CHT sorption on 3D-printed nanocellulose meshes further enhanced the performance, resulting in a high modulus and tensile strength compared to pure nanocellulose samples. The CHT-sorbed nanocellulose mesh demonstrated non-cytotoxicity towards human monocytes/macrophages.

These studies highlight the potential of combining CHT with other polymers or materials to enhance hydrogels’ mechanical properties and 3D printability for various applications in tissue engineering and biomedical fields.

## 3. 3D Printing of Aerogels

Aerogels are 3D porous materials with a lightweight structure, high surface area, porosity, heat and mechanical properties stability, customisable surface characteristics, and practical sorption abilities. These can be derived from inorganic sources like silica, alumina, and titania or organic sources such as resorcin-formaldehyde, melamin-formaldehyde, and various polysaccharides like cellulose, starch, lignin, chitin, chitosan, alginate, carrageenan, pectin, agarose, and agar, as well as proteins [96,97,98,99,100]. These aerogels are formed through a post-polymerization process where the solvent trapped in the gel structure is replaced by a gas, like air, resulting in a mesoporous structure with a significant amount of space (95–99%) [100,101,102]. Aerogels have already found applications in diverse fields like biomedicine, pharmaceuticals, nutrition, the food industry, oil-water separation, air filtration, metal-ion absorption, dye adsorption, thermal insulation, CO_2_ capture, catalysis, and electrochemistry, among others [96,97,98,99,100,102,103,104,105,106,107,108,109]. Various technologies have been developed to produce 3D functional aerogels, including AM techniques like extrusion or DIW, inkjet printing, and stereolithography. This process involves synthesising ink, 3D printing, drying, and post-processing to obtain aerogels with desired shapes [8,110].

The three primary methods employed are extrusion-based printing (EBP), inkjet-based printing (IBP), and SLA [8,110]. Among these, EBP is the most commonly used technique for 3D printing aerogels. It involves using paste-like inks that exhibit shear-thinning properties, extruded through a nozzle as filaments. These filaments maintain their shape due to their sufficient strength. Typically, achieving this requires high solid content in the inks or adding agents that modify the ink’s rheology. The compatibility of inks allows for the straightforward implementation of EBP by depositing viscoelastic ink through a nozzle. The viscoelastic behaviour of EBP inks is usually characterised by a plot that illustrates the storage and loss moduli in relation to shear stress. When shear stress surpasses the yield point (gelation), *G*′ becomes lower than *G*″ (*G*″/*G*′ > 1), indicating a primarily viscous (liquid-like) behaviour of the ink. At low shear stress values, the storage modulus becomes higher than the loss modulus (*G*″/*G*′ < 1), indicative of an elastic (solid-like) behaviour [8,110]. Once gels with the desired shapes are 3D printed, an appropriate drying method, such as supercritical (sc) fluid drying, freeze-drying, or ambient temperature drying, is employed to obtain aerogels. Finally, post-processing steps are implemented to enhance the structural integrity and functionality of aerogels [8].

Freeze-drying and drying at ambient temperatures most frequently lead to the destruction of the pore structure. Although they are sometimes referred to as aerogels in the literature, the terminology is controversial in the aerogel community, as some argue that aerogels are dried with scCO_2_, cryogels are obtained by freeze-drying, and xerogels are dried at ambient temperature. According to the IUPAC definition (“Gel comprised of a microporous solid in which the dispersed phase is a gas.”), however, there is no distinction in this sense, so in the present review, we group all as aerogels according to the literature references [111,112].

Overall, 3D-printed aerogels provide access to customised structures and complex geometries with extremely low density and high porosities. However, there is a limited availability of suitable printable aerogel materials with optimal mechanical properties and processability; therefore, achieving high resolution is a challenge. The required post-processing steps pose additional difficulty in predicting the final properties. Future research should focus on material development to improve mechanical properties and increase printing resolution without compromising structural integrity.

### 3.1. Cellulose

CNCs have garnered considerable attention because of their high Young’s modulus, high strength, lightweight nature, low density, sustainability, biocompatibility, biodegradability, recyclability, and abundant availability [113,114,115,116,117]. As a result, it is plausible and advantageous to replace conventional thermoplastics used in 3D printing with cellulosic materials such as CNCs. There is a growing emphasis on producing purely cellulosic aerogels using microfibrillated cellulose (MFC), CNFs, or CNCs [113,118,119,120,121,122,123,124]. Usually, the straightforward freeze-drying method is implemented as it enables the creation of aerogels with low density and high porosity [113].

**Figure 5:**
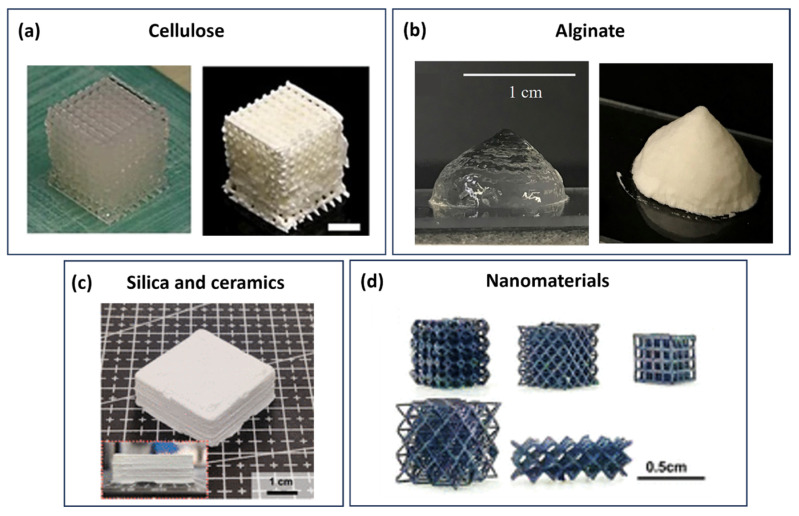
Example of 3D-printed aerogels from cellulose, alginate, silica, ceramics, and nanomaterials, respectively. (**a**) DIW 3D-printed aerogel using 20 wt.% CNC gel. Adapted with permission from Li et al. [113]. Copyright 2017, Scientific Reports. (**b**) The 3D-printed structures with 3% GEL-ALG and a G/A ratio of 2:1 before and after freeze-drying. Adapted with permission from Kuo et al. [157]. Copyright 2021, Elsevier. (**c**) Supercritically dried 3D PAS aerogel. Adapted with permission from Wang et al. [168]. Copyright 2021, American Chemical Society. (**d**) Optical images of 3D-architected MXene aerogel. Adapted with permission from Yang et al. [190]. Copyright 2022, American Chemical Society.

In 2017, Li et al. [113] reported DIW printing of pure CNC aerogels, aiming to achieve complex structures and customised inner pore architectures. The unique dual-pore structures of CNC aerogels hold the potential for the effective integration of cells, which is essential for tissue engineering applications. Careful management of the aerogel’s CAD, the gel formulations, and DIW processing parameters, including the nozzle tip size, was necessary to control the inner pore architecture and overall structural customisation precisely. Various CNC hydrogel compositions with concentrations of 11.8, 15, 20, and 30 wt.% were prepared and, using the DIW approach in 3D, printed into a simple 1 cm^3^ cubic structure. The viscosity of the CNC gel was also assessed using cone and plate viscometer tests, revealing an increase in viscosity with a higher CNC concentration. Moreover, the CNC gel transitioned from shear-thickening to shear-thinning behaviour as the CNC weight percentage increased. This transition was confirmed by changes in the concavity of the shear stress versus shear rate curve, indicating that high shear forces could induce a certain level of CNC alignment with increased shear rate and CNC concentration. This shear-thinning effect was beneficial for efficient gel deposition during DIW. After deposition, the gel’s high viscosity helped maintain the printed features. Different pressures were required for gel deposition depending on the gel’s viscosity. By optimising the deposition pressure, printing speed, G-code layer height, and extrusion width, it was possible to control the height and width of the gel filament while printing each combination of CNC gel concentration and nozzle tip size. The resulting printed gel structures closely resembled the CAD-designed structures, thus showcasing the ability to print CNC gel structures from CADs directly. Additionally, no noticeable shrinkage or damage to the aerogels occurred after freeze-drying (Figure 5a), likely attributable to the high Young’s modulus and strong hydrogen bonding properties of the CNCs.

In 2018, Li et al. [125] pursued a similar approach to explore CNF instead of CNCs as the primary component for aerogel production. They focused on creating deformable networks through physical entanglements and hydrogen bonding. The authors modified TEMPO and high-pressure homogenisation to produce defibrillated and T-CNF gels suitable for 3D printing via DIW. Achieving stable and uniform CNF suspensions with adequate viscosity and fluid dynamic properties was crucial for DIW printing pure CNF gels. TEMPO oxidation of CNF increased the negative repulsive charge between fibres, which could be counteracted by high-pressure homogenisation [126,127,128,129,130]. The viscosity of CNF suspensions increased with the number of homogenisation passes due to enhanced fibre-fibre interactions through defibrillation. However, increasing the number of homogenisation passes initially increased and then decreased the viscosity of T-CNF suspensions. The viscosity of the gel played a significant role in maintaining the shape of 3D-printed structures after DIW processing. A T-CNF gel with a viscosity of 5.15 × 10^−1^ ± 6.00 × 10^−3^ Pa·s at the highest shear rate of 500 s^−1^ (at 2.8 wt.% concentration) exhibited shear-thinning behaviour, enabling the printing of complex structures while retaining their shape. After freeze-drying, the 3D-printed T-CNF aerogel structures also served as templates for incorporating additional functionality.

In 2019, Kam et al. [131] reported on the 3D printing of cellulose-based aerogels for various applications such as wound healing, scaffolds, lightweight foams, and aerogel substrates for supercapacitors [132,133,134,135,136,137,138]. The authors employed direct cryowriting (DCW), a method that allows simultaneous printing and freezing, to create bioinspired structures using rod-shaped CNCs and xyloglucan (XG) as a binder [139,140,141,142]. Low-temperature deposition manufacturing (LDM) processes were employed for rapid ink fixation to achieve the desired shape retention. The authors prepared aerogels using aqueous mixtures of CNCs (4 wt.%) and XG through 3D printing with DCW at cryogenic temperatures, followed by post-processing through freeze-drying.

As mentioned, extrusion-based 3D printing requires ink compositions with shear-thinning rheological properties for effective object printing. CNCs exhibit shear-thinning behaviour and relatively high viscosities at low shear and low concentrations, making them suitable for water-based 3D printable inks [143,144,145]. Adding XG to CNCs resulted in shear-thinning behaviour for all samples, with higher XG concentrations leading to higher viscosities. However, the gel structure collapsed at high XG concentrations (1:4 and 1:10 XG:CNC) at high shear rates. Pure XG solutions at 4 wt.% exhibited Newtonian behaviour, indicating unaffected viscosity under shear stress. The increase in CNC viscosity with the addition of XG can be attributed to the binding of XG to CNCs [146,147]. At low concentrations, XG acts as a cross-linking agent. In contrast, it exerts a slippery effect at high concentrations, mimicking its role in the plant cell wall and acting as a rheological modifier. 

In 2021, Feng et al. [148] obtained 3D-printed polyimide (PI) composite aerogels for heat management applications using water-based inks prepared with CNCs as renewable nanofillers. The ink primarily consisted of water-soluble polyamic acid ammonium salts (PAAS) formed by the complexation of polyamic acids and triethylamine (TEA). CNCs were included in the PAAS aqueous solutions to create physically crosslinked hydrogels suitable for DIW. Freeze-drying was employed after printing to create porous structures. The authors investigated the effect of CNCs on the rheological behaviour of PAAS/CNC inks, varying the CNC concentration from 5.6 wt.% to 7.4 wt.% while keeping the polyamic acid concentration at 7.0 wt.%. All inks demonstrated significant shear-thinning behaviour, with the viscosity of the ink using 6.5 wt.% CNCs diminishing at high shear rates, allowing for high flow rate extrusion. The performance of DIW also depended on the storage modulus and yield stress, where high *G*′ and yield stress were desired for successful stacking and unsupported part spanning. All inks exhibited gel-like behaviour with *G*′ greater than *G*″ at low shear stress and transitioned into a liquid-like state (*G*″ > *G*′) at high shear stress due to CNC alignment [149]. The strength of the physical networks in the inks increased with CNC concentration, while adding more polyamic acid did not improve ink printability.

More recently, in 2022, Zhang et al. [150] used Fe_3_O_4_ nanoparticles for organic dye molecules as a nanoprobe in fabricating magnetic cellulose-based aerogels through freeze-drying for organic adsorption [151]. A lattice-like structural hydrogel was built using a magnetic ink with a moderate rheology suitable for direct printing. Crosslinking with an epichlorohydrin (ECH) aqueous solution increased ink viscosity and strength. The ink crosslinked for 36 h displayed higher *G*′ and viscosity than the ink crosslinked for 0 h, enabling filament shape retention in 3D printing. The magnetic ink’s rheological behaviour and crosslinking time could be readily adjusted, allowing for the creation of designed 3D patterns. The preparation of the designed 3D patterns is controlled by several factors: the rheological properties of the ink, the diameter of the needle, printing speed, and printing pressure. By controlling the crosslinking time, the cellulose-based ink could be tailored to exhibit self-thickening and self-strengthening properties with adequate storage modulus and viscosity.

Additionally, in 2022, Zhou et al. [152] presented findings on the fabrication of 3D-printed structures made of CNF inks. They achieved this by adjusting the flow properties and printing parameters and exploring the resulting materials in thermal insulation and energy storage applications. After freeze-drying, these 3D-printed CNF structures were transformed into porous, independent, and thermally insulating CNF frameworks, displaying superior properties. Three viscoelastic inks were formulated using CNF concentrations of 1.5%, 3%, and 4% through concentration and homogenisation to create these structures. The relationship between viscosity and CNF concentration was positive, indicating that higher CNF loading facilitated the formation of a robust interconnected network. The CNF inks also exhibited noticeable shear-thinning behaviour, which can be attributed to the disruption of the network structure and the alignment of CNFs along the direction of shear. This characteristic was advantageous for extruding the ink smoothly through a small nozzle under printing pressure [144,153]. According to the power-law model, the values of *n* were consistently less than 1, suggesting that the CNF inks possessed the characteristic of shear-thinning as a typical non-Newtonian fluid [154]. To investigate the thixotropic behaviour of the CNF inks, changes in viscosity were continuously monitored by alternating low and high shear rates. Initially, a low shear rate of 10^−2^ s^−1^ was applied for 3 min to simulate pre-extrusion conditions. Then, the shear rate was rapidly increased to 10^2^ s^−1^ to mimic the ink extrusion through a tiny nozzle. The researchers observed that the viscosity promptly decreased due to the disruption of the CNF gel network when subjected to a high shear rate, consistent with the shear-thinning behaviour. Significantly, when the shear rate returned to 10^−2^ s^−1^, the viscosity recovered to its initial values almost fully within a short period. These shear-thinning and thixotropic properties ensured smooth ink extrusion and shape retention of the printed filament. Furthermore, the viscoelastic properties of the CNF inks were evaluated through dynamic rheological measurements, considering the effects of oscillatory stress and angular frequency. The results demonstrated a predominantly solid-like behaviour of the CNF inks, as indicated by the significantly higher value of *G*′ compared to *G*″ in the region below the yield stress (stress at which *G*′ = *G*″). However, when the stress exceeded the yield stress, the ink exhibited liquid-like behaviour (*G*′ < *G*″) due to the breakdown of the gel network structure [155]. Interestingly, the *G*′ in the plateau region (where *G*′ remains nearly constant regardless of stress) and the yield stress were linearly proportional to the CNF concentration. The ink with a CNF concentration of 4 wt.% exhibited substantially higher *G*′ and G* across the entire range of angular frequencies. These elevated values of *G*′, yield stress, and G* ensured structural integrity during the printing process while preserving the desired shape fidelity after 3D printing.

### 3.2. Alginate

ALG bioinks are widely employed for creating 3D frameworks using various 3D printing techniques due to their minimal toxicity and straightforward crosslinking capability. The production of bioactive 3D hydrogel frameworks using biocompatible materials characterised by a complex internal structure comprising mesoporous and macroporous features, along with an extensively interconnected porosity, holds significant importance in the field of BTE, among other related applications [156].

In 2021, Kuo et al. [157] reported 3D printable GEL/ALG hybrid hydrogels with varying total solid concentrations (3%, 5%, and 7%) and GEL/ALG ratios (1:2, 1:1, and 2:1), which were subsequently processed through freeze-drying to solidify the matrices and enhance their shelf stability post-3D printing. Alginate hydrogel exhibits notable pliability and a liquid-like nature. Consequently, it is commonly combined with various other biopolymers, including starch, gelatin, and cellulose derivatives, to create a hybrid gel [158,159]. Regarding the rheological properties, the pure GEL group exhibited shear-thinning behaviour, with the apparent viscosity decreasing as the shear rate increased. Meanwhile, the apparent viscosity of pure ALG remained fairly constant as the shear rate increased. Based on the 3D printing outcomes and rheological data, utilising pure GEL and ALG was not feasible. Both pure GEL and ALG exhibited higher apparent viscosity and stress under the same shear rate, with an increase in the total solid concentration. The 3%, 5%, and 7% GEL-ALG matrix formulations with GEL/ALG ratios of 2:1, 1:1, and 1:2 all exhibited shear-thinning behaviour. In this study, the yield stress was estimated by determining the stress at the point where *G*′ declined by 10% of the *G*′ within the LVER. Under the same GEL and ALG ratio, an increase in the total solid concentration resulted in a significant increase in yield stress. In terms of viscoelasticity, the results of the oscillation frequency sweep test indicated that, with the exception of 3% GEL/ALG with ratios of 1:1 and 1:2, a *G*′ higher than the *G*″ trend was observed. The tan *δ* parameter represents the ratio of *G*″ to *G*′ in viscoelastic materials. In the case of tan *δ* < 1, a decrease in plasticity and an increase in elasticity are observed. The 3% GEL/ALG with ratios of 2:1 (Figure 5b) and 1:1, 5% GEL/ALG with ratios of 2:1 and 1:1, and 7% GEL/ALG with ratios of 2:1, 1:1, and 1:2 all exhibited tan *δ* values ranging from 0.22 to 0.93, indicating a more solid-like nature of these materials. Additionally, a loss factor (Equation (6)) in the range of 0.48–0.58 during the frequency sweep of 15–40 rad/s was determined for the printable materials demonstrated, which corresponds to the frequency associated with the 3D printing settings. In contrast, 3% GEL/ALG with a ratio of 1:2 and 5% GEL/ALG with a ratio of 1:2 exhibited tan *δ* > 1, indicative of a more liquid-like property. Thus, optimal printing quality was observed for 3% GEL/ALG with a ratio of 2:1, 5% GEL/ALG with a ratio of 1:1, and 7% GEL/ALG with a ratio of 1:2 hybrid hydrogels, which exhibited suitable extrudability and shape retention performance. The semi-solid objects produced via 3D printing underwent a freeze-drying process, yielding a porous, low-density matrix characterised by crispiness along with reduced moisture content and water activity. These materials can be used as delivery systems for incorporating other bioactive components in bioengineering, pharmaceuticals, nutraceuticals, and the food industry.

In 2022, Iglesias-Mejuto et al. [156] reported ALG hydrogel-based scaffolds using extrusion-based 3D-printing technology. This approach aimed to preserve a complex nanostructure through the utilisation of scCO_2_ drying, resulting in the fabrication of 3D-aerogel-based scaffolds characterised by structural integrity and customised designs. However, ALG scaffolds possess weaknesses for treating bone defects due to their limited bioactivity [160]. Therefore, HA was employed as a filler in composite BTE scaffolds owing to its high cell adhesion and bioactivity, its strong reabsorption ability, and its overall biocompatibility [161,162]. The ALG-HA aerogel scaffolds for BTE were dual-crosslinked through CaCl_2_ gelation, hydrogel ageing, and glutaraldehyde (GA) crosslinking on the aerogel structures. Regarding rheological behaviour, under rest-like conditions, *G*″ exceeded *G*′ for all ink formulations, indicating a major viscous component. This liquid-like behaviour favours the extrusion-based 3D printing method [163]. The moduli of the inks containing HA were significantly higher, demonstrating the reinforcement effect of this inorganic compound on the ink’s mechanical properties. Under high shear strain conditions, both moduli experienced a rapid decrease, particularly *G*′. This effect, more pronounced than in *G*″, suggested that the physical entanglement between ALG chains was diminished with the chains aligning in the direction of stress, which facilitates the extrusion process. Upon removal of the strain, the ink regained its initial *G*′ and exhibited the same *G*″ as at rest, indicating a fast recovery of the initial entanglements. This behaviour is necessary for maintaining the desired shape after the extrusion of the ink. Flow tests confirmed the pronounced shear-thinning behaviour of all inks (with and without HA) under low-shear-stress conditions. This feature should ease the extrusion process at moderate pressures [164]. Incorporating HA improved the printability of the ink compared to those without HA. However, when the HA content reached 24 wt.%, the printability of the inks decreased primarily due to the printer’s difficulty in extruding and the increased strength of ALG hydrogels when higher HA concentrations were used. Another possible reason is the chemical reaction between the Ca^2+^ ions on the HA surface and the -COO^−^ groups of the alginate, resulting in ionic bonding that enhances the strength of the structures. Ultimately, osteoinductive ALG-HA aerogel scaffolds were successfully produced [165].

### 3.3. Silica and Ceramics

Silica aerogels are considered the most extensively investigated and applied within the aerogel’s spectrum. Compared to silica aerogels, AM offers an alternative for downsizing since they face challenges in achieving intricate designs and precise shapes. DIW demonstrates superiority in printing aerogels due to its customisable printer mode, CAD printing path, and fine ink compatibility. Two prerequisites must be met: the silica ink should possess optimal printing rheology and be capable of solidifying through a chemical reaction. The inks should exhibit shear-thinning properties to guarantee smooth extrusion and provide self-support after printing [7,166,167,168,169,170,171,172,173,174,175]. Therefore, in 2020, Zhao et al. [166] introduced a DIW approach to producing miniaturised silica aerogel objects using a silica aerogel powder slurry in a diluted silica nanoparticle suspension (sol). The printed gel could also be made hydrophobic before solvent removal through scCO_2_ drying. The ink’s rheology could be adjusted according to the application, with higher viscosity being adequate for open structures with overhangs and wider spans and lower viscosity for seamless merging of filaments into continuous membranes without voids. Miniaturised silica aerogels can be used in thermal insulation, electrical, magnetic, optical, chemical, and medical applications.

More recently, in 2021, Wang et al. [168] reported 3D-printed silica aerogels using aerosil powders (PAS) (Figure 5c) and silica aerogel powders (PAG) to obtain silica aerogel insulation materials. Considering the rheological requirements for printing and the aerogel composition, nanosilica powders proved to be ideal rheological control agents for silica inks due to their specific shear response in dispersion [176,177]. After printing, the high *G*′ value aided in preserving the complex structures of printed objects, enabling subsequent hydrophobic modification, solvent replacement, and scCO_2_ drying processes.

Ceramic aerogels show promise in various fields, such as thermal insulation, catalysis, filtration, environmental remediation, and energy storage, among others. However, their brittleness, caused by the inadequate bonding between oxide ceramic nanoparticles, limits conventional shaping and post-processing methods [110]. In 2022, Wang et al. [110] developed a versatile thermal-solidifying direct-write assembly technique for creating ceramic aerogels using DIW. By formulating the ink, the interaction of hydrogen bonds between fumed silica and PVA polymers facilitated the formation of interconnected networks, resulting in highly printable and moldable inks. Inks containing nanoscale ceramics (boehmite and rutile) exhibited shear-thinning behaviour similar to pseudoplastic fluids, enabling the AM of multi-component ceramic aerogels. The controlled solidification of ceramic inks was achieved by catalysing the thermal decomposition of ammonia derived from urea. This approach allowed ceramic aerogels to maintain structural integrity and shape fidelity through solvent exchange and scCO_2_ drying processes.

### 3.4. Nanomaterials—New Developments

Novel materials such as graphene oxide (GO), MXenes, carbon nanotubes, and titanium dioxide (TiO_2_) are revolutionising several industries due to their advanced properties and versatile applications.

Graphene, a 2D material, possesses low density, favourable mechanical properties, a large surface area, and good electrical conductivity. Chemically derived GO-based aerogels, known for their uncomplicated synthesis methods and ability to achieve diverse pore structures, are the most commonly documented 3D graphene structures [142]. In a study conducted by García-Tuñón et al. [178] in 2017, GO’s use of water-based pastes for robocasting enabled the printing of various materials like polymers, ceramics, and steel. Due to its amphiphilic nature and 2D structure, GO serves as a dispersant, viscosifier, and binder. During the investigation, GO suspensions in water exhibited shear-thinning properties. It is widely recognised that the viscosity and viscoelasticity of GO suspensions in water heavily rely on their concentrations [179,180,181,182]. In 2020, Masud et al. [183] introduced a graphene-biopolymer aerogel preparation method using a DIW 3D printing technique followed by freeze-drying, specifically for water treatment applications. In 2022, Chandrasekaran et al. [184] demonstrated the application of DIW technology for fabricating 3D catalytic electrodes in electrochemical applications. Specifically, they employed DIW to construct a hierarchical 3D catalyst framework by combining a highly porous graphene aerogel with commercial MoS2 powders, freeze-drying, and annealing to reduce GO. The rheological behaviour of GO dispersions has been extensively studied to enable the construction of graphene oxide into more intricate designs [182].

DIW showcases significant potential in constructing 3D electrode structures for incorporation into energy storage systems [155,185,186,187,188,189]. The 2D transition metal carbides, nitrides, and carbonitrides (MXenes) are emerging as promising materials for high-performance energy storage devices. These materials possess attractive attributes such as favourable electrical conductivity, high-density packing, and abundant surface functional groups [190,191,192,193]. Hydrophilicity is imparted to MXene through oxygenated and halogenated surface functional groups (T_x_), allowing the formation of aqueous suspensions for electrode fabrication using techniques such as vacuum filtration, freeze-drying, and spin coating [194,195,196,197]. Nonetheless, challenges remain in designing the electrode architecture [155,190,198].

MXene exhibits limited gelation capability primarily regulated by electrostatic interaction, posing difficulties in fabricating robust 3D MXene aerogels [192,194,199]. Numerous endeavors have been undertaken to fabricate 3D structures utilizing MXene via EBP methods, emphasizing the pivotal significance of regulating ink rheology in this process. Several methodologies have been documented and employed to fulfil the specified criteria or demands [153,190,200,201,202].

In 2022, Dai et al. [198] showcased a 3D printing technique to manufacture lightweight 3D MXene frameworks with excellent structural design flexibility through DIW. They developed a highly printable aqueous ink by modifying the MXene ink rheology using GO microgels. The durable and flexible ink mixture can construct self-supporting 3D macroscopic structures in ambient conditions using a 3D printer with a three-axis control system. Post-printing, these structures undergo liquid nitrogen treatment to preserve their well-defined shapes. Through a subsequent freeze-drying process, porous filaments emerge with sustained structural integrity and connectivity. The honeycomb-like cellular MXene/GO scaffold attains conductivity and transforms into robust frameworks following treatment at 80 °C and 500 °C under an argon atmosphere. Similarly, in 2022, Yang et al. [190] developed additive-free aqueous MXene inks with a wide range of concentrations (5 to 150 mg mL^−1^) and low viscosity (*η* > 2.5 × 10^−3^ Pa·s), taking advantage of cation-induced self-gelation of MXene ink in a 3D printed resin template. Leveraging the structural design flexibility provided by digital light processing (DLP) 3D printing technology, the researchers effectively manufactured intricately designed 3D MXene structures characterised by low tortuosity and high mass loading. The application of electrodes was envisioned for the 3D-architected MXene aerogels, achieved through the freeze-drying process of MXene hydrogels (Figure 5d). In a similar context, in 2022, Zhou et al. [155] reported various TEMPO-mediated oxidised CNFs with different diameters and varying surface carboxylate groups. These CNFs were explored as rheology modifiers in combination with other functional materials to create 3D printable gel inks [203,204,205]. The authors carefully controlled the oxidant content during the TEMPO oxidation process to achieve CNFs with specific properties. At a low concentration of 8 wt.%, these CNFs were used as rheology modifiers to develop viscoelastic MXene-based inks; subsequently, the freeze-dried 3D architectures formed self-standing, hierarchically porous 3D electrodes, exhibiting excellent capacitive performance.

Highly porous lightweight carbon aerogels possess distinct characteristics such as low density, elevated surface area-to-volume ratio, and superior strength-to-mass ratio. Carbon nanotubes (CNTs) have found extensive utility due to their elongated structure, exceptional electrical conductivity, elevated specific surface area (SSA), and mechanical rigidity. One method for producing CNT aerogels involves employing isolated CNTs in water with a dispersing agent, allowing for customisation of shape and size without compromising intrinsic properties. Particularly, individual dispersed single-walled carbon nanotube (SWCNT) rods can easily form rigid networks by means of attractive forces resulting from van der Waals interactions, attributable to their small diameter and significant length [206,207,208,209]. In a recent study of 2021 by Jeong et al. [206], GEL-CNT wet-gels were introduced as promising ink materials, offering scalability and ease of processing. Gelatine was selected as a component due to its environmentally friendly nature and helical structure held together by hydrogen bonds, which can be modified with temperature changes, known as the sol-gel transition. Furthermore, the gelatine’s zwitterionic structure enhances its affinity towards CNT surfaces [210,211,212]. The GEL-CNT ink was extruded efficiently through a nozzle while maintaining the CNT network to achieve aerogels with the desired shape. Subsequently, the extruded GEL-CNT wet-gels were subjected to scCO_2_ drying and then pyrolysed to obtain graphene-coated CNT (Gr-CNT) aerogels. Ultimately, the GEL-CNT wet-gels’ increased yield stress and shear-thinning behaviour make them suitable for scalable AM applications.

Inks without additives, similar to traditionally casted aerogels, require specific formulations to utilise nanomaterials’ intrinsic or combined properties in a 3D-printed aerogel that is dried using scCO_2_. In 2022, Rebber et al. [213] introduced a 3D printing technique for producing aerogels based on TiO_2_. The researchers developed additive-free inks of gelled TiO_2_ nanoparticles with a volumetric concentration of 4 vol%. Instead of incorporating rheological additives, the low particle concentration was compensated by printing the ink in a liquid bath with an alkaline pH. Furthermore, the ink formulation allowed for easy processing of multi-component inks by combining TiO_2_ with other nanoparticle dispersions, such as spherical Au nanoparticles (AuNP) or Au nanorods (AuNR), before gelation. The final nanoparticle-based aerogels can be applied in photothermal devices and can be envisioned for other applications.

These materials offer unique properties and contribute significantly to developing more efficient, sustainable, and innovative technologies across multiple sectors, leading to material science and technological advancements.

## 4. Conclusions and Future Directions

In this review article, it was possible to verify the importance of rheological properties for the product development of hydrogels and aerogels through 3D printing. Throughout the revision, it was found that rheology techniques needed to be easier to access and widely implemented. Therefore, more investment must be made to make the devices increasingly portable and adapted to an industrial reality. Polymeric hydrogels play a crucial role in creating scaffolds by means of 3D printing. However, there is a constrained assortment of viable hydrogels that can be formulated into polymer-based inks, as it remains challenging to fine-tune their properties. In the field of aerogels, there is also space to advance and optimise this type of material, which is very promising for many areas due to its properties. Hence, 3D-printed aerogels still have some limitations that must be overcome. These include resolution and printing speed, scaling up while maintaining consistency, mechanical strength and durability of material properties and structure, a limited choice of materials suitable for 3D-printed aerogels, and cost efficiency. To overcome these limitations, continuous research and development efforts are needed to refine material compositions, printing technologies, and post-processing techniques and explore new applications to take 3D-printed aerogels to the next level. In recent years, aerogels have witnessed substantial development and were even classified by IUPAC in 2022 as one of the Top Ten Emerging Technologies in Chemistry. The definition of aerogels remains open to discussion and clarification, especially regarding the drying methods, as evidenced by ongoing deliberations within the aerogel’s community.

From a material perspective, cellulose and alginate are among the most studied biomaterials that have been explored, both in hydrogels and aerogels. Comparing the hydrogel with the aerogel version of these materials, distinct physical states, properties, and applications make them suitable for different purposes. Cellulose and alginate hydrogels, with their soft and flexible nature, are particularly suitable for biomedical applications, while aerogels, with their lightweight and porous structure, excel in areas like thermal insulation and lightweight structural components. In any application, the rheological properties of the respective inks are crucial for the success of their 3D printing. Furthermore, an appropriate drying method must be employed to go from hydrogel to aerogel. Post-processing steps can also enhance their structural integrity and functionality.

Generally, a printable ink intended for 3D printing must possess excellent shear-thinning characteristics, allowing for smooth ink extrusion through the deposition nozzle when subjected to shear forces and forming seamless filaments. Furthermore, it should demonstrate favourable thixotropic properties that enable the rapid recovery of viscosity or viscoelasticity following the printing process. Additionally, the ink should possess an appropriate yield stress and *G*′ to ensure high-fidelity preservation in the printed 3D structures, effectively preventing deformation or collapse. There are several strategies to address the challenges and limitations of 3D printing, namely adjusting printing parameters that help improve adhesion between layers and reduce defects, developing or selecting materials specifically tailored to 3D printing processes to address limitations in material properties, introducing post-processing methods, quality control, computer-aided simulation and modelling, and training professionals involved in 3D printing processes. Implementing these remedial strategies can help mitigate the identified problems in 3D printing and improve the quality, reliability, and performance of printed structures. Furthermore, the interaction between the substrate of the print bed and the printing material during 3D printing underlines the need for comprehensive studies aimed at elucidating and optimising the role of the print bed substrate to improve the quality and performance of 3D printed objects. Few references in the literature emphasise this factor as a parameter to be studied and evaluated, as most of the materials are printed on glass substrates. These properties significantly influence the ink’s printability, encompassing its extrudability, filament formation, shape accuracy, and geometrical precision.

In conclusion, future research should focus on scalability using rheological techniques, which requires theoretical and computational investigation, as well as studies regarding the interaction between the substrate of the print bed and the printing material, as well as the development of new ink formulations with nanomaterials for the production of advanced aerogels.

## Data Availability

No data were used for the research described in this article. The papers used are cited and given as references.
